# Modelling the epidemiologic impact of achieving UNAIDS fast-track 90-90-90 and 95-95-95 targets in South Africa

**DOI:** 10.1017/S0950268818003497

**Published:** 2019-03-01

**Authors:** N. N. Abuelezam, A. W. McCormick, E. D. Surface, T. Fussell, K. A. Freedberg, M. Lipsitch, G. R. Seage

**Affiliations:** 1 William F. Connell School of Nursing, Boston College, 140 Commonwealth Avenue, Chestnut Hill, MA 02467, USA; 2 Department of Epidemiology, Harvard T.H. Chan School of Public Health, 677 Huntington Avenue, Boston, MA 02115, USA; 3 Divisions of General Internal Medicine and Infectious Disease, the Medical Practice Evaluation Center, Massachusetts General Hospital, 55 Fruit Street Boston MA 02114, USA; 4 Division of Infectious Disease, Brigham and Women's Hospital, 75 Francis Street Boston, MA 02115, USA; 5 Department of Health Policy and Management, Harvard T.H. Chan School of Public Health, 677 Huntington Avenue, Boston, MA 02115, USA; 6 Center for Communicable Disease Dynamics and Department of Immunology and Infectious Diseases, Harvard T.H. Chan School of Public Health, 677 Huntington Avenue, Boston, MA 02115, USA

**Keywords:** Agent-based models, HIV disease (AIDS), mathematical modelling, South Africa

## Abstract

UNAIDS established fast-track targets of 73% and 86% viral suppression among human immunodeficiency virus (HIV)-positive individuals by 2020 and 2030, respectively. The epidemiologic impact of achieving these goals is unknown. The HIV-Calibrated Dynamic Model, a calibrated agent-based model of HIV transmission, is used to examine scenarios of incremental improvements to the testing and antiretroviral therapy (ART) continuum in South Africa in 2015. The speed of intervention availability is explored, comparing policies for their predicted effects on incidence, prevalence and achievement of fast-track targets in 2020 and 2030. Moderate (30%) improvements in the continuum will not achieve 2020 or 2030 targets and have modest impacts on incidence and prevalence. Improving the continuum by 80% and increasing availability reduces incidence from 2.54 to 0.80 per 100 person-years (−1.73, interquartile range (IQR): −1.42, −2.13) and prevalence from 26.0 to 24.6% (−1.4 percentage points, IQR: −0.88, −1.92) from 2015 to 2030 and achieves fast track targets in 2020 and 2030. Achieving 90-90-90 in South Africa is possible with large improvements to the testing and treatment continuum. The epidemiologic impact of these improvements depends on the balance between survival and transmission benefits of ART with the potential for incidence to remain high.

## Introduction

Countries around the world have been tasked by UNAIDS with reaching the fast-track 90-90-90 targets by 2020 and the 95-95-95 targets by 2030 [1]. These targets aim to have 90% of people living with human immunodeficiency virus (PLHIV) diagnosed, 90% treated among those diagnosed and 90% virally suppressed among those treated with antiretroviral therapy (ART) by 2020. This goal would result in 73% overall suppression of PLHIV [2], while UNAIDS 2030 targets of 95-95-95 by 2030 would result in 86% overall suppression.

UNAIDS developed these targets as aspirational goals for countries with presumed impact on the epidemiology of HIV infection worldwide. The potential impact of achieving these goals on the global epidemiology of HIV infection is unknown [3–5]. Additionally, little is known about what types of testing, treatment, and prevention interventions will allow countries to achieve these UNAIDS targets by 2020. It is possible that interventions which allow for the rapid achievement of these goals may not have sustained epidemiologic impact. Conversely, combinations of interventions that do not achieve the 90-90-90 or 95-95-95 targets may still have long-term impact on the epidemiology of HIV infection and may vary by strategy.

We aim to identify the incremental changes to the current testing and treatment policies in South Africa that would lead to the achievement of the 90-90-90 and 95-95-95 fast-track targets using the HIV Calibrated Dynamic Model (HIV-CDM). We also aim to understand the epidemiologic impact of these changes on HIV prevalence, incidence and infections averted between 2014 and 2030. We compare the impact of combinations of interventions that help to achieve fast-track targets and combinations that have sustained impact on HIV prevalence, incidence and infections averted in South Africa.

## Methods

### HIV-CDM model description

The HIV-CDM is an individual-based stochastic mathematical model that simulates transmission of HIV in a population of male and female persons allowed to interact with one another based on partnership rules previously described [6]. Individuals are deemed either low- or high-risk based on the frequency of new sexual partnership formation and the number of concurrent partnerships. The force of infection in the model varies with the HIV-RNA of the infected partner, condom use and circumcision status of the male partner. The model is calibrated to historical South African prevalence, incidence and sexual behaviour and validated with real data to represent the South African epidemic in the post-calibration period. The three-phase model calibration resulted in 564 parameter sets that produced dynamics consistent with historical data in South Africa, with each assigned a likelihood weight quantifying fit to historical HIV prevalence data [6]. Individual and population level data are attainable from the HIV-CDM at monthly intervals, and all epidemiologic outcomes (HIV prevalence, incidence and infections averted) are determined directly from the model output.

### Testing and treatment continuum

The HIV-CDM structure allows PLHIV to pass through the testing and treatment continuum provided by the Cost Effectiveness of Preventing AIDS Complications International (CEPAC-I) model and the impact of various testing and treatment assumptions on epidemic dynamics can thereby be examined [7, 8]. HIV-RNA is stochastically assigned by CEPAC-I and, in the absence of treatment, determines the monthly decline in CD4 count, which in turn leads to increased risks of opportunistic infections and HIV-related mortality [7, 9]. Individuals receiving ART have reductions in their HIV-RNA, which in turn reduces their HIV transmission potential and CD4 decline. In the testing continuum, individuals access care and are offered an HIV test based on clinical symptoms. A proportion of individuals accept HIV tests and a proportion are linked to care if the test is positive. When individuals link to care and subsequently meet the CD4 ART start threshold (Supplementary Table S1), they enter the treatment continuum in CEPAC-I. Individuals begin treatment according to treatment availability, which depends on the rate at which ART is expanded (see below). Those receiving ART have a probability of HIV-RNA suppression; for those who reach suppression, a monthly probability of late ART failure is assigned [10]. Additionally, individuals can be lost to follow-up at a specified yearly rate (Supplementary Table S1) [11, 12].

### Interventions

We allow for changes in the following aspects of the testing and treatment continuum: rate of intervention availability; HIV testing interval offered as part of a test and treat programme; HIV test acceptance proportion; proportion of tested individuals who link to treatment; proportion of individuals on treatment who are suppressed at 6 months; proportion of individuals on suppressive ART who virologically fail; proportion of individuals on ART who are lost to follow-up and CD4 threshold for initiation of ART. We chose these parameters for this analysis as they each reflect the stages of the testing and treatment continuum that can be influenced by policy initiatives and changes to the current prevention landscape in South Africa and ultimately on the achievement of the fast-track targets. Current levels of access and values for each of these parameters were found in the literature and denoted as the baseline levels (Table 1).

**Table 1.** Testing and treatment input changes made for each of the scenarios examining immediate (in 2015) incremental improvements to baseline and targeted interventions for a model of HIV transmission in South Africa

Scenario	Targeted group	Rate of testing, treatment and prevention intervention availability	HIV testing interval	HIV test accept rate (%)	Linkage to care (%)	ART supp. at 6 mo. (%)	Monthly ART late fail prob. (%)	LTFU on ART (%)	CD4 threshold for treatment in 2015
Baseline		Slow	1 year	50	47	78	0.1	9.9	<500/μl
30% improvement on baseline		Slow/medium/rapid	8 months	65	65	84	0.07	7	All CD4
50% improvement on baseline	N/A	Slow/medium/rapid	6 months	75	75	89	0.05	5	All CD4
80% improvement on baseline	N/A	Slow/medium/rapid	3 months	90	90	96	0.02	2	All CD4
**Age targeting**									
17–24 year olds 100% improvement	17–24 year olds		1 month	100	100	100	0	0	All CD4
	25–99 year olds	Slow	1 year	50	47	78	0.1	9.9	<500/μl
17–24 year olds 50% improvement	17–24 year olds		6 months	75	75	89	0.05	5	All CD4
	25–99 year olds	Slow	1 year	50	47	78	0.1	9.9	<500/μl
17–29 year olds 100% improvement	17–29 year olds		1 month	100	100	100	0	0	All CD4
	30–99 year olds	Slow	1 year	50	47	78	0.1	9.9	<500/μl
30–59 year olds 100% improvement	30–59 year olds		1 month	100	100	100	0	0	All CD4
	17–29 and 60–99 year olds	Slow	1 year	50	47	78	0.1	9.9	<500/μl
**Risk group targeting**									
Low risk (LR) females 100% improvement	LR females		1 month	100	100	100	0	0	All CD4
	Non-LR females	Slow	1 year	50	47	78	0.1	9.9	<500/μl
LR males 100% improvement	LR males		1 month	100	100	100	0	0	All CD4
	Non-LR males	Slow	1 year	50	47	78	0.1	9.9	<500/μl
Commercial sex workers (CSW) 100% improvement	CSW		1 month	100	100	100	0	0	All CD4
	Non-CSW	Slow	1 year	50	47	78	0.1	9.9	<500/μl
High risk (HR) females 100% improvement	HR females		1 month	100	100	100	0	0	All CD4
	Non-HR females	Slow	1 year	50	47	78	0.1	9.9	<500/μl
HR females 50% improvement	HR females		6 months	75	75	89	0.05	5	All CD4
	Non-HR females	Slow	1 year	50	47	78	0.1	9.9	<500/μl
HR males 100% improvement	HR males		1 month	100	100	100	0	0	All CD4
	Non-HR males	Slow	1 year	50	47	78	0.1	9.9	<500/μl
HR males 50% improvement	HR males		6 months	75	75	89	0.05	5	All CD4
	Non-HR males	Slow	1 year	50	47	78	0.1	9.9	<500/μl

ART, antiretroviral therapy; HIV, human immunodeficiency virus; LTFU, loss to follow-up; Prob., probability; Supp., viral suppression; LR, low risk; HR, high risk.

### Scenarios of treatment availability growth and testing/treatment parameter improvement

Various countries, including South Africa, will likely take steps to increase the availability and uptake of particular testing and treatment interventions over time to ensure that quality of care is maintained. In our modelling simulations, we consider two dimensions of improvements: (1) growth in treatment availability and (2) improvement in testing and treatment cascade parameters.

(1) We consider three rates at which access to HIV treatment is expanded from baseline levels over time: slow, medium and rapid. When demand exceeds supply in any scenario, priority is given to those most in clinical need. The proportion of the population that can receive treatment each year corresponding to each of these expansion rates can be found in Supplementary Table S2.

(2) We consider three scenarios for improvement of the testing and treatment cascade, with 30%, 50% and 80% improvements occurring instantaneously in 2015. For example, a scenario with 30% improvement refers to a situation in which, from 2015 onwards, dropout or failure at each of the testing and treatment stages (described in the Interventions section) is reduced instantaneously by 30% from its baseline level (Table 1). We consider all nine combinations of improvements in treatment availability growth and cascade efficiency.

Additionally, scenarios are examined in which testing and treatment interventions are targeted at particular demographic or at-risk populations. By maintaining baseline values for all testing and treatment parameters for the general population and changing the testing and treatment parameters for the targeted population, we simulate targeted interventions. The targeted subgroups of the population we considered included women or men, young (17–29 year old and subgroups) or older (30–59 year old) individuals, high or low-risk individuals, commercial sex workers and combinations of some of these groups (i.e. young high-risk men). Some scenarios assume that the testing and treatment continuum for these targeted subgroups are perfected, while other scenarios assume that the continuum are improved by a specified percentage (Table 1).

### Simulation details

Simulations shown are initialised with 100 000 persons and six initial cases of HIV introduced among high-risk men and women. Intervention scenarios are implemented in 2015 (with baseline levels in testing and treatment occurring prior to 2015) and simulations are run forward through 2030. Results shown for a particular scenario represent the weighted median and weighted interquartile range (IQR) of the 564 parameter sets that comprise the top 90% of the weight to fit from the original calibration run [6]. Incidence is defined as the number of infections per 100 person-years (P-Y). The HIV-CDM is coded in C++.

## Results

We aim to compare predictions from the HIV-CDM to data collected in South Africa on the percentage of PLHIV in South Africa that were on treatment and suppressed. In 2012 in South Africa, 37.8–55.0% of all PLHIV were diagnosed, and it was estimated that overall viral suppression among PLHIV was 23.8–25.0% [1, 13]. Simulations were created in which the historical testing and treatment continuums in South Africa were simulated according to published proportions. This allowed us to compare ART uptake and suppression in the model with data from South Africa. In our simulation runs in 2012, 41.7% (IQR: 40.0–43.5%) of PLHIV were diagnosed, and we predicted overall suppression of 24.0% (IQR: 21.8–26.1%) among PLHIV in the model (Supplementary Table S3). A continuation of baseline levels would result in a 27.0% (IQR: 25.6–28.6%) increase in prevalence and a 6.8% (IQR: 4.7–8.7%) increase in incidence by 2030 from 2015 levels. These baseline policies would yield 44.4% (IQR: 43.8–45.0%) of PLHIV suppressed in 2020 and 47.2% (IQR: 46.8–47.6%) suppressed in 2030.

### Scenarios

Of all nine scenarios considered, only moderate and rapid treatment expansion combined with 80% improvement in testing and treatment achieved 90-90-90 targets in 2020 (Table 2). The 95-95-95 targets are achieved in 2030 when testing and treatment parameters are improved by 80%, regardless of intervention availability timing. Improving the testing and treatment continuum by 80% with medium intervention availability results in 82.6% (IQR: 81.6, 83.5%) overall suppression in 2020, 86.4% (IQR: 85.9, 86.9%) overall suppression in 2030, dramatic declines in incidence (to 0.80 cases per 100 P-Y, IQR: 0.71, 0.91 in 2030) and slight decreases in prevalence (to 24.6%, IQR: 22.4, 27.0% in 2030) from 2015 levels (Supplementary Fig. S1). Higher overall suppression is achieved in 2030 than in 2020 in all incremental scenarios. The proportion of PLHIV suppressed in 2030 is about the same for rapid and slow rollout of the 30%, 50% and 80% improvement scenarios likely due to the fact that more individuals are lost to follow-up in the rapid scenario over time than in the slow and medium scenarios despite more individuals on treatment early in the simulation in the rapid scenario (Supplementary Fig. S2).

**Table 2.** Results for scenarios examining impact on 90-90-90 targets and epidemiologic outcomes of incremental changes to baseline testing and treatment cascades and targeted scenarios on specific demographic and at-risk groups

Scenario	% Infections averted (2015 to 2030) compared to baseline	% Life months gained (2015 to 2030) compared to baseline	% Deaths averted (2015 to 2030) compared to baseline	HIV prevalence among 17+ in 2030 (%)	Annual HIV incidence in 2030 among 17+ (cases per 100 P-Y)	Total % of HIV + supp. in 2020	Total % of HIV + supp. in 2030
Baseline	N/A	N/A	N/A	33.2 (30.2, 37.1)	2.70 (2.36, 3.21)	44.4 (43.8, 45.0)	47.2 (46.8, 47.6)
**30% improvement on baseline**							
Slow rollout	21.2 (19.9, 22.6)	0.6 (0.5, 0.7)	3.5 (3.1, 3.8)	29.1 (26.5, 32.4)	1.73 (1.54, 1.96)	60.5 (54.5, 62.7)	66.1 (65.5, 66.7)
Medium rollout	24.8 (23.5, 26.0)	1.1 (1.0, 1.2)	4.7 (4.5, 5.0)	28.9 (26.4, 31.8)	1.74 (1.51, 1.96)	63.6 (62.9, 64.3)	66.2 (65.6, 66.7)
Rapid rollout	25.9 (24.3, 27.6)	1.2 (1.1, 1.4)	5.1 (4.7, 5.6)	28.8 (26.4, 31.6)	1.72 (1.51, 1.94)	64.2 (63.7, 64.6)	66.1 (65.6, 66.7)
**50% improvement on baseline**							
Slow rollout	28.2 (26.1, 30.1)	0.8 (0.6, 0.9)	4.7 (4.1, 4.9)	27.7 (25.3, 31.1)	1.37 (1.22, 1.54)	62.2 (55.7, 67.8)	73.8 (73.2, 74.4)
Medium rollout	34.4 (32.9, 35.8)	1.4 (1.3, 1.6)	6.5 (6.1, 6.8)	27.2 (25.0, 30.0)	1.36 (1.21, 1.53)	71.1 (70.3, 71.9)	73.9 (73.4, 74.5)
Rapid rollout	37.2 (35.0, 39.3)	1.8 (1.6, 2.1)	7.3 (6.7, 8.0)	27.0 (24.9, 29.6)	1.37 (1.21, 1.53)	72.4 (72.0, 72.9)	73.9 (73.4, 74.4)
**80% improvement on baseline**							
Slow rollout	37.3 (32.7, 40.4)	1.0 (0.7, 1.1)	5.7 (4.9, 6.1)	26.1 (23.6, 29.8)	0.83 (0.73, 0.96)	63.7 (57.3, 69.4)	85.8 (85.0, 86.7)
Medium rollout	49.0 (47.3, 50.6)	1.9 (1.7, 2.0)	8.4 (8.0, 8.9)	24.6 (22.4, 27.0)	0.80 (0.71, 0.91)	82.6 (81.6, 83.5)	86.4 (85.9, 86.9)
Rapid rollout	55.7 (53.2, 57.9)	2.5 (2.2, 2.8)	10.0 (9.3, 10.9)	23.8 (22.0, 25.9)	0.81 (0.72, 0.89)	85.6 (85.2, 85.9)	86.4 (86.0, 86.9)
**Age targeting**							
17–24 year olds 100% improvement	59.6 (57.9, 61.6)	0.9 (0.8, 1.0)	5.1 (4.8, 5.4)	20.2 (18.3, 22.2)	0.52 (0.44, 0.61)	68.5 (66.8, 70.1)	76.7 (75.1, 78.1)
17–24 year olds 50% improvement	36.5 (34.6, 38.4)	0.7 (0.6, 0.8)	4.0 (3.8, 4.3)	25.5 (23.5, 27.9)	1.33 (1.20, 1.48)	62.6 (61.4, 63.9)	69.2 (68.4, 69.9)
17–29 year olds 100% improvement	68.1 (65.8, 69.8)	1.2 (1.1, 1.4)	6.6 (6.2, 7.1)	19.0 (17.3, 20.8)	0.37 (0.31, 0.45)	76.2 (74.7, 77.5)	83.4 (82.2, 84.5)
30–59 year olds 100% improvement	17.3 (15.3, 19.6)	1.3 (1.1, 1.6)	5.0 (4.2, 5.8)	30.9 (28.2, 34.3)	2.03 (1.79, 2.36)	69.2 (68.0, 70.4)	73.3 (72.3, 74.3)
**Risk group targeting**							
Low risk (LR) females 100% improvement	12.2 (10.0, 14.7)	1.1 (0.9, 1.3)	4.6 (3.8, 5.3)	32.0 (29.3, 35.5)	2.36 (2.06, 2.75)	65.7 (63.4, 68.9)	62.0 (60.5, 63.8)
LR males – 100% improvement	9.5 (6.0, 13.9)	0.7 (0.5, 1.0)	3.1 (2.3, 4.3)	31.9 (29.3, 35.0)	2.40 (2.11, 2.74)	59.6 (56.7, 62.9)	59.3 (57.1, 61.4)
Commercial sex workers (CSW) 100% improvement	0.5 (−0.1, 1.0)	0.04 (0.02, 0.1)	0.2 (0.1, 0.3)	32.9 (30.1, 37.0)	2.66 (2.31, 3.18)	45.5 (44.8, 46.3)	48.4 (47.8, 49.0)
High risk (HR) females 100% improvement	8.3 (6.4, 10.0)	0.4 (0.3, 0.6)	2.0 (1.3, 2.7)	31.8 (28.8, 35.5)	2.39 (2.05, 2.83)	54.5 (50.3, 57.6)	58.0 (53.5, 61.3)
HR females 50% improvement	4.5 (3.3, 5.7)	0.3 (0.2, 0.4)	1.4 (0.8, 1.9)	32.4 (29.5, 36.4)	2.55 (2.17, 3.03)	50.3 (47.9, 52.6)	53.3 (50.8, 55.1)
HR males 100% improvement	50.2 (45.0, 54.0)	0.7 (0.6, 0.9)	4.0 (3.4, 4.6)	21.7 (19.8, 25.2)	0.95 (0.80, 1.22)	59.6 (55.6, 63.3)	65.0 (61.2, 68.8)
HR males 50% improvement	26.4 (23.6, 28.3)	0.5 (0.4, 0.6)	2.5 (2.1, 3.0)	27.3 (24.9, 30.7)	1.77 (1.54, 2.13)	53.6 (50.9, 55.9)	56.2 (54.0, 58.2)

HIV, human immunodeficiency virus; Supp., viral suppression; HR, high risk; LR, low risk; P-Y, person-years.

On average, the sexually active population accounts for 61% of the total population size in 2020 and 62% in 2030 across scenarios. HIV prevalence among those sexually active (17+ years of age) at the start of 2015 for all runs is 26.0% (23.9, 28.8). Annual HIV incidence among those sexually active at the start of 2015 for all runs is 2.54 (2.19, 3.01) cases per 100 P-Y. Numbers represent the weighted median and IQR, in parentheses, for each quantity.

A large reduction in incidence occurs in the first year after the introduction of new programmes in the medium and rapid scenarios (Supplementary Fig. S1). The slow rollout scenario produces consistent reductions to incidence and then plateaus at different years based on the improvement strength. Despite stochasticity, incidence is consistent across intervention availability speeds. HIV prevalence is higher in 2030 for the slow intervention availability scenarios than in the medium and rapid scenarios. The proportion of infections averted, proportion of life months gained and proportion of deaths averted increases with the speed of intervention availability.

### Targeting scenarios

When targeting young people between the ages of 17 and 29 years old with perfect testing and treatment, nearly all of the epidemiologic impact can be attributed to putting 17–24 year olds on suppressive therapy. Improving upon baseline by 50% in the 17- to 24-year-old group results in progress towards achieving the fast-track targets by 2030 (69.2% overall suppression proportion, IQR: 68.4–69.9%) but falls short of the target. Reductions in incidence can be very high if all 17–24 year olds go through the testing and treatment campaign when optimised (Table 2, note the rest of the population experiences baseline level interventions). Likewise, when we improve the testing and treatment continuum for 17–29 year olds we observe the largest reductions in prevalence due to the reduction in transmission in this highly sexually active group. Additionally, among the targeted scenarios we observe a large proportion of deaths averted when targeting 17–29 year olds (6.6%, IQR: 6.2–7.1%) and percent of life months gained (1.2%, IQR: −1.1, 1.4%).

When targeting high-risk groups with extensive testing and treatment programmes, major impact is made on HIV prevalence, incidence and infections averted, but little progress is made towards achieving the fast-track goals. In the initiation of the simulation runs, high-risk males make up from 7 to 40% of the male population and 5–40% of the female population (Supplementary Table S1). Improving the continuum for high-risk males has the largest impact on incidence and prevalence of any of the risk-targeting policies considered, with absolute reductions in incidence to 0.95 cases per 100 P-Y (IQR: 0.80, 1.22) and absolute reductions in prevalence to 21.7% (IQR: 19.8, 25.2%) in 2030 (Table 2). The total proportion suppressed in the population is 65.0% (IQR: 61.2, 68.8%), well below the 86% fast-track targets in 2030 (Table 2). Targeting high-risk females, low-risk females and commercial sex workers decreases incidence but has a minimal impact on prevalence; prevalence, continues to increase over time driven by increases in survival.

## Discussion

The speed at which testing and treatment are made available to the entire HIV-infected population in South Africa impacts the reductions in modelled prevalence. Faster expansion and/or higher success through the continuum results in slightly greater reductions to prevalence. Higher success through the continuum reduces incidence, but faster expansion has little impact on reduction in incidence. Incidence remains relatively high in 2030 (0.80 cases per 100 P-Y, IQR: 0.71, 0.91 for 80% improvement, medium rollout), despite achievement of fast-track targets. Importantly, achievement of the fast-track targets in both 2020 and 2030 are not possible with 30% and 50% reductions in loss at each stage of the testing and treatment continuum. A continuation of current HIV testing and treatment policies (baseline levels) would not result in South Africa achieving UNAIDS fast-track targets by either 2020 or 2030, despite increasing levels of virally suppressed individuals over time. Substantial improvements (80%) in reducing losses at each stage of the HIV testing and treatment continuum would be needed to meet fast-track targets by 2020 with medium or rapid availability of interventions to the general population.

It is important to note that targeting interventions to specific age groups may be an effective, more logistically feasible, and less costly alternative to targeting individuals based on risk behaviour. Targeting testing and treatment to young people who are sexually active results in shifting the epidemic trajectory and reducing incidence – a significant challenge globally [14]. Ironically, controlling and eliminating transmission within high-risk groups may be at odds with achieving the UNAIDS goals of high levels of overall testing and treatment coverage, as can be seen by the low levels of viral suppression in the general population (Table 2). When targeting risk groups, targeting high-risk males with many sexual partners is the most effective strategy to lower incidence and reduce prevalence, but does not result in achievement of the fast-track targets. The reduction in incidence is attributed to the large number of high-risk men, their partners and their sexual acts each month. Targeting high-risk males may be the most attractive approach in resource poor settings with limited access to ART, but identification and engagement of high-risk males could prove difficult [15].

Cluster randomised trials are currently underway in Sub-Saharan Africa to estimate the impact of combination prevention programmes on HIV incidence. While these trials are important to understanding the implementation challenges in expanding testing and treatment efforts in high incidence settings, their results are limited in scope by potential power and cost issues. Agent-based models allow for the simulation of counterfactuals and therefore allow for comparisons only attempted by randomised controlled trials with little unmeasured confounding [16]. Agent-based models help us understand the potential long-term consequences of large-scale improvements to testing and treatment in various international settings. They also allow us to examine a wide-range of outcomes for a large number of scenarios, something that cannot be achieved in a community-randomised trial setting.

The conclusions of this modelling study are limited by the assumptions made in the model structure. The HIV-CDM models only heterosexual transmission; it does not account for testing and treatment benefits for mother to child transmission, transmission from perinatally infected adolescents or individuals infected through injection drug use. We have also assumed that sexual behaviour does not change with wide scale testing and treatment, an assumption that may not be correct [17]. Heterogeneity in testing behaviour and in response to treatment is expected but is not explored in detail in this analysis. Further, implementing multiple concurrent interventions that require action by individuals (i.e. frequent and repetitive HIV testing) in the population may result in participant fatigue or non-motivated participation, which is not factored into the analysis. We also implemented testing and treatment interventions at idealised levels, which may not be feasible for all levels of the continuum in real-world populations.

Countries have begun to implement changes that would help them achieve fast-track targets in recent years. The Botswana Combination Prevention Program recently showed that Botswana is close to reaching the 90-90-90 goals well ahead of 2020. They report 83% diagnosed, 87% treated and 96% suppression in the general HIV-infected population, resulting in an overall 70% suppression rate [18]. Uganda has recently shown that it is possible to achieve up to 80% ART initiation within 2 weeks of diagnosis using novel recruitment methods [19]. In Zambia and South Africa, 71% of individuals enrolled in the PoPART trial have initiated ART in 2015 [20]. The long-term epidemiologic consequences of these achievements will likely be seen in the coming years as these trials continue. In the meantime, agent-based models, like the HIV-CDM, are able to highlight the expected epidemiologic consequences and help power these trials.

The UNAIDS fast-track 90-90-90 and 95-95-95 targets aim to motivate policy makers to undertake large scale changes to the testing and treatment landscape to improve individual clinical outcomes. In countries that achieve these targets, there is the potential for large-scale improvements in incidence and prevalence. We have shown that targeted testing and treatment interventions have the potential to make epidemiologic changes without achieving the fast-track targets. The extent of these shifts, and the potential for wide-scale impact, depends largely on the rate at which interventions are made available to the HIV-infected population in South Africa. Increased funding should be made available to help countries achieve these targets and increase the rate of intervention rollout. Achieving the fast-track targets is possible by both 2020 and 2030 if local public health organisations and departments are able to sufficiently improve upon the efficacy and efficiency of the testing and treatment continuum.

## Supplementary material

For supplementary material accompanying this paper visit https://doi.org/10.1017/S0950268818003497. click here to view supplementary material
