# Supplementary material for: Modelling the epidemiologic impact of achieving UNAIDS fast-track 90-90-90 and 95-95-95 targets in South Africa
Source: Epidemiol Infect. 2019 Mar 1;147:e122. doi: 10.1017/S0950268818003497 (PMC6452860; doi:10.1017/S0950268818003497)
Supplement: Supplementary file 1 [file S0950268818003497sup001.docx]

| **Parameter** | **Value** | **Reference** |
| --- | --- | --- |
| Population size at model initiation | 100,000 |  |
| Number of births per person per month | 0.0022 | ([1](#_ENREF_1)) |
| Proportion of the population who is male | 0.50 | ([1](#_ENREF_1)) |
| Age of first sex | 17 years | ([2-4](#_ENREF_2)) |
| Proportion of newborn males circumcised | 0.35 | ([2](#_ENREF_2), [3](#_ENREF_3), [5](#_ENREF_5), [6](#_ENREF_6)) |
| Proportion of females becoming CSW | 0.01-0.04** |  |
| Proportion of males in the HR group | 0.07-0.4 |  |
| Proportion of non-CSW females in the HR group | 0.01-0.3** |  |
| ***Sexual Partnership Characteristics*** |  |  |
| *Steady Partnerships* |  |  |
| Duration of partnership^b^ | 10.20 years (SD 7.80 years) | ([7-9](#_ENREF_7)) |
| Number of sex acts per partnership per month^c^ | 9 | ([10-12](#_ENREF_10)) |
| Probability of male condom use per sexual act^d^ (%) | 12 (SD 6) | ([4](#_ENREF_4), [13](#_ENREF_13), [14](#_ENREF_14)) |
| *Regular Partnership* |  |  |
| Duration of partnership^b^ | 13.50 mo. (SD 9 mo.) | ([2](#_ENREF_2), [15](#_ENREF_15)) |
| Number of sex acts per partnership per month | 4-11** |  |
| Probability of male condom use per sexual act (%) | 29 (SD 15) | ([2](#_ENREF_2), [4](#_ENREF_4), [16](#_ENREF_16), [17](#_ENREF_17)) |
| *Casual Partnership* |  |  |
| Duration of casual partnership | 1 sexual act | (MA) |
| Number of sex acts per partnership per month^c^ (MA) | 1 |  |
| Probability of male condom use per sexual act^d^ (%) | 37 (SD 19) | ([2](#_ENREF_2), [4](#_ENREF_4), [16](#_ENREF_16)) |
| *CSW Encounters* |  |  |
| Duration of CSW encounter | 1 sexual act | (MA) |
| Number of sex acts per partnership per month^c^ | 1 | (MA) |
| Probability of male condom use per sexual act^d^ (%) | 50 (SD 25) | ([13](#_ENREF_13)) |
| ***Partnership Selection Criteria*** |  |  |
| Average number of years younger the female is compared to male partner (steady, regular and casual)^b^ | 5 (SD 2.50) | ([7](#_ENREF_7), [14](#_ENREF_14)) |
| Average number of years younger CSW is compared to male partner^b^ | 13 (SD 6·50) | ([18](#_ENREF_18), [19](#_ENREF_19)) |
| ***Sexual Network Parameters*** |  |  |
| High-risk multiplier | 1-10** |  |
| High-risk multiplier CSW | 30-100** |  |
| Partner acquisition multiplier while in steady partnership or low-risk males | 0-1** |  |
| Assortativeness parameter for steady, regular, and casual partnerships | 0.2-0.8** |  |
| ***Intervention Efficacy*** |  |
| Circumcision efficacy (%) | 56 | ([20-23](#_ENREF_20)) |
| Condom efficacy (%) | 80 | ([24](#_ENREF_24)) |
| ***HIV Testing*** |  |  |
| Intervention HIV testing interval | 1 year | ([25](#_ENREF_25), [26](#_ENREF_26))(MA) |
| Average background HIV test frequency | Every 10 years | ([26](#_ENREF_26)) |
| Sensitivity of HIV test (%) | 99.9 | (MA) |
| Specificity of HIV test (%) | 99.4 | (MA) |
| Sensitivity in acute phase^a^ of HIV test (%) | 0 | (MA) |
| Linkage to care (%) | 46.8 | ([26](#_ENREF_26)) |
| Mean initial CD4 cell count, cells/μl |  |  |
| Acute, primary^a^ HIV infection | 884 | ([27](#_ENREF_27)) |
| ***ART*** |  |  |
| CD4 ART start criteria in 2013 | <350 cells/µl | ([28](#_ENREF_28)) |
| ART Suppression at 6 months (%) | 78 | ([29](#_ENREF_29)) |
| Monthly late ART failure probability (%) | 0.1 | ([29](#_ENREF_29)) |
| ART program loss to follow-up at 12 mo. (%) | 9.9 | ([30](#_ENREF_30), [31](#_ENREF_31)) |
| ***Natural History*** |  |  |
| Mean monthly CD4 decline (cells/μl) by HIV RNA level |  | ([32](#_ENREF_32), [33](#_ENREF_33)) |
| >30,001 copies/ml | 6.4 |  |
| 10,001-30,000 copies/ml | 5.4 |  |
| 3,001-10,000 copies/ml | 4.6 |  |
| 501-3,000 copies/ml | 3.7 |  |
| <500 copies/ml | 3.0 |  |
| Probability of transmission per sexual act by HIV RNA (copies/ml) |  | ([11](#_ENREF_11), [12](#_ENREF_12), [34](#_ENREF_34), [35](#_ENREF_35)) |
| 0-500 | 0.0001 |  |
| 501-3,000 | 0.0012 |  |
| 3,001-10,000 | 0.0012 |  |
| 10,001-30,000 | 0.0014 |  |
| 30,001 + | 0.0023 |  |
| Acute infection^a^ | 0.0082 |  |
| Late-stage infection^a^ | 0.0036 | ([12](#_ENREF_12)) |

**Abbreviations:** ART= antiretroviral therapy; CSW= commercial sex worker; HIV=human immunodeficiency virus; HR=high risk; MA = Model assumption; mo.=month.

**^a^** Acute infection is the first three months post-infection; chronic infection immediately follows primary infection and continues until the individual’s CD4 count drops below 50/mm^3^; late-stage infection occurs when the individual’s CD4 drops below 50/mm^3^.

^b^ The duration of partnerships and the average number of years between partners are parameters chosen from a normal distribution with the denoted mean and standard deviation.

^c^ The number of sex acts per partnership per month parameter is chosen from a Poisson distribution with the denoted mean (and standard deviation).

^d^ The probability of a condom use in each partnership parameter is chosen from a beta distribution. This distribution is converted to a normal distribution with the denoted mean and standard deviation for presentation in table.

** These parameters were varied in the calibration procedure. Each of the 564 parameter sets has a randomly selected value from within these ranges for each of the 12 varied parameters.

**Supplemental Table 2. Rollout Specifications.** This table indicates the population size and historical treatment rollout numbers in South Africa from 2002-2014. Because the model population size is smaller than that of the actual South African population, a treatment availability ratio is calculated from these numbers which is used to scale the model population size to determine the number of treatment slots available in the model run. Specifically, the historical number being treated is divided by the total population size for each year to determine the historical treatment ratio. This ratio is then multiplied by the model population size in the same year to determine the number of available treatment slots for that year. After 2014, assumptions are made about the speed of ART rollout (slow, medium, or rapid) by changing the treatment availability ratio, which is similarly multiplied by the model population size in the same year to determine the number of available treatment slots. The number of individuals on treatment is dictated by the number of individuals who have gotten through the treatment cascade for which there is an available treatment slot.

| **Year** | **Population Size** (in millions) ([36](#_ENREF_36)) | **Historical Number Infected** (in millions) ([37](#_ENREF_37)) | **Historical Number Being Treated** ([37](#_ENREF_37), [38](#_ENREF_38)) | **Historical Treatment Ratio** | **Rapid Treatment Availability Ratio** | **Medium Treatment Availability Ratio** | **Slow Treatment Availability Ratio** |
| --- | --- | --- | --- | --- | --- | --- | --- |
| *2002* | 40.00 | 4.68 | 15000 | 0.0004 |  |  |  |
| *2003* | 41.00 | 4.95 | 26000 | 0.0010 |  |  |  |
| *2004* | 41.80 | 5.15 | 55000 | 0.0010 |  |  |  |
| *2005* | 42.50 | 5.32 | 206718 | 0.0050 |  |  |  |
| *2006* | 43.40 | 5.46 | 324754 | 0.0070 |  |  |  |
| *2007* | 44.60 | 5.59 | 458951 | 0.0100 |  |  |  |
| *2008* | 45.90 | 5.72 | 730183 | 0.0160 |  |  |  |
| *2009* | 47.40 | 5.82 | 971556 | 0.0200 |  |  |  |
| *2010* | 48.80 | 5.88 | 1389865 | 0.0280 |  |  |  |
| *2011* | 50.10 | 5.97 | 1702060 | 0.0340 |  |  |  |
| *2012* | 52.60 | 6.07 | 2150881 | 0.0410 |  |  |  |
| *2013* | 52.98 | 6.30 | 2596020 | 0.0490 |  |  |  |
| *2014* | 54.00 | 6.50 | 2754000 | 0.0510 |  |  |  |
| *2015* |  |  |  |  | 0.06 | 0.06 | 0.06 |
| *2016* |  |  |  |  | 0.99 | 0.08 | 0.07 |
| *2017* |  |  |  |  | 0.99 | 0.12 | 0.08 |
| *2018* |  |  |  |  | 0.99 | 0.20 | 0.09 |
| *2019* |  |  |  |  | 0.99 | 0.36 | 0.10 |
| *2020* |  |  |  |  | 0.99 | 0.68 | 0.11 |
| *2021* |  |  |  |  | 0.99 | 0.68 | 0.12 |
| *2022* |  |  |  |  | 0.99 | 0.68 | 0.13 |
| *..* |  |  |  |  | .. | .. | .. |
| *2030* |  |  |  |  | 0.99 | 0.80 | 0.21 |

**Supplemental Table 3. Comparison of HIV-CDM to South African data in 2012**. Model results (weighted median and quartiles) in 2012 are compared with data from South Africa in 2012. Indicators including HIV prevalence, HIV incidence, proportion of HIV-positives who are diagnosed, and proportion of HIV-positive who are virally suppressed are documented below.

| **Indicator** | **HIV-CDM Model Prediction** | **South African Data** |
| --- | --- | --- |
| **HIV prevalence (%) in 2012** | 24.9 (22.9, 27.4) | 18.8 (17.5, 20.3)* ([25](#_ENREF_25)) |
| **Women** | 28.2 (25.8, 30.5) | 23.2 (21.3, 25.1)* (25) |
| **Men** | 21.6 (19.2, 24.3) | 14.5 (12.8, 16.3)* (25) |
| **HIV incidence (cases per 100 person-years) in 2012** | 2.59 (2.23, 3.06) | 1.72 (1.38, 2.06)* ([25](#_ENREF_25)) |
| **Women** | 2.92 (2.55, 3.42) | 2.28 (1.84, 2.74)* (25) |
| **Men** | 2.28 (1.89, 2.74) | 1.21 (0.97, 1.45)* (25) |
| **% of HIV-positives who are diagnosed** | 44.1 (42.1, 46.1) | 37.8-55.0 ([25](#_ENREF_25), [39](#_ENREF_39), [40](#_ENREF_40)) |
| **% of HIV-positives who are virally suppressed** | 28.6 (26.0, 31.0) | 23.8-25.0 ([39](#_ENREF_39), [40](#_ENREF_40)) |

*** Represents data from those 15-49 years of age.**

**Supplemental Table 4. Historical ART Treatment Guidelines.** Assumptions for access to ART are highlighted in the table for each of the years in which a major treatment guideline was revised. We assumed that the first two years of ART distribution were dominated by individuals with severe OIs. We then followed the treatment guidelines published by the South African Department of Health for the subsequent years until present day. Treatment decisions in 2015 are dictated by Table 1.

| **Year** | **Treatment Guidelines** | |
| --- | --- | --- |
|  | ***WHO Stage/OI*** | ***CD4*** |
| ***2002*** | 4 | Irrespective |
|  | 3 | Irrespective, including recurrent or persistent oral thrush and recurrent invasive bacterial infections |
| ***2004*** ([41](#_ENREF_41)) | 4, 3 | Irrespective |
|  | 1,2,3 | <200/mm^3^ |
| ***2010*** ([42](#_ENREF_42)) | TB | <350/mm^3^ |
|  | 4 | Irrespective |
|  | Irrespective | <200/mm^3^ |
| ***2013*** ([43](#_ENREF_43)) | 3,4 | Irrespective |
|  | Irrespective | <350/mm^3^ |

**Supplemental Figure 1. Graphical representation of prevalence and incidence.** This figure shows the changes in the weighted median prevalence (solid, left axis) and incidence (dashed, right axis) for the scenarios that improve upon the Baseline. The population size is increasing in all scenarios. In the Baseline scenario the total model population size increases from 367,438 to 415,748 from 2020 to 2030.

**Supplemental Figure 2. Loss to follow-up for 80% improvement scenarios.** This figure shows the proportion of those on ART that are lost to follow-up over time for the 80% improvement scenarios (rapid, medium, and slow rollout). The figure highlights the fact that more individuals are lost to follow-up over time in the rapid scenario than the slow scenario, influencing the proportion suppressed in 2030.

**Supplemental Information**

In addition to the details found in the text, we refer the reader to (Abuelezam et al. PLoS One 2014) for details of the model calibration procedure and the sexual partnership structure.

**Sexual Partnership Network Details**

Partnerships between male and female individuals in the model are formed and dissolved every month based on a number of different partnership characteristics including length of the partnership, partnership type, and the number of sexual acts each month. Males chose females for partnerships based on her partnership (single or non-single) and CSW status applying the user-defined partner selection weights for each partnership type. Men chose from eligible age categories for a female based on the user-defined average age difference for that partnership type. Durations of partnerships are drawn from distributions defined in Table S1. The HIV-CDM tracks the type and the number of partnerships each individual forms each month allowing males and females to engage in multiple partnerships within a given month. An individual in a steady partnership has a decreased acquisition rate for other partnership types. Once all males have formed new partnerships for a given month, all sexual acts between partners occur and the model determines if HIV transmission has occurred for each sexual act.

The probability of HIV transmission at each sexual act with an HIV infected individual (PTrAct) in the HIV-CDM is dependent upon the following parameter values: the *probability of transmission per act* (β_HVL_), which is dependent on an infected individual’s HIV RNA and stage of HIV infection (see Table S1); the *per-act probability of condom use by partner type* (con_p_); the *protective efficacy of a condom* *(*protEffCon); *circumcision status* (circ_i_); and the *protective efficacy of circumcision* (protEffCirc). PTrAct increases as the HIV RNA level increases and it is decreased by circumcision and condom use. Therefore, the PTrAct for HIV-infected males transmitting to an uninfected female (MtoF) is:

PTrAct_MtoF_ = β_HVL_*[1-(con_p_*protEffCon)]

and PTrAct for HIV-infected females transmitting to an uninfected male (FtoM) is:

PTrAct_FtoM_ = β_HVL_*[1-(con_p_*protEffCon)]*(1-Circ_i_*protEffCirc).

From this, the probability of transmission per month (PTrMonth) for each partnership is calculated using the following equation:

PTrMonth = 1-(1-PTrAct)^n^

where n is the number of acts per partnership per month and PTrAct is either MtoF or FtoM depending on which partner is infected.

**Calibration**

We have described the three phases of the model calibration below. The goal of the calibration procedure was to produce model runs that replicated data on sexual behavior, partnership histories, and HIV prevalence in South Africa.

**Phase I – Behavioral Calibration**

The goal of the first calibration step is to determine whether a parameter set produces realistic patterns of sexual partnerships and acts, based on prior knowledge and an extensive literature review of South African data. This calibration step is necessary because many model inputs that affect partnership prevalence and sexual behavior (i.e. partnership formation rates) have not been directly measured in the literature, but many model outputs (e.g. the proportion of the population in particular partnership types) have been measured and reported. Acceptable ranges (comparable to uniform priors) were specified for five behavioral outputs, and then an additional four constraints were imposed in the form of inequalities between male and female behavioral parameters. We set to zero the weights of parameter sets that produced model output that fell outside these limits, for **any one of more** of the constraints, effectively discarding these parameter sets. The parameter sets whose output fell within all constraints were considered to pass “phase 1” or “behavioral” calibration.

Phase 1 calibration restricted the model output using pre-specified prior distributions, of the following quantities:

- The proportion of the **entire sexually active (SA) population** that was in a steady partnership (non-single) the year prior to Phase 1 calibration (the last year of model initialization)
- The proportion of **SA males** that had at least one casual partnership the year prior to Phase 1 calibration (the last year of model initialization)
- The proportion of **SA males** that had at least one CSW partnership the year prior to Phase 1 calibration (the last year of model initialization)
- The proportion of **SA** **males** with more than 1 partner (one of which was a steady/regular partnership) the year prior to Phase 1 calibration (the last year of model initialization)
- The average number of acts per person among the **entire SA population** in the last month of model initialization

In addition, there were conditions specific to sexually active females only:

- The casual partnership prevalence among sexually active females could be **no greater than** that of sexually active males.
- The percent of sexually active females in multiple partnerships within the past month could be **no greater than** that of sexually active males.
- The ratio of acts per month for low risk sexually active females could be **no greater than** that of high risk females, multiplied by some user-defined ratio.

For the analyses reported here, these user-defined ratios were all set to one.

Phase 1 calibration examined model output after the model initialization period (currently set to 50 years). If the partnership outcomes did not satisfy all of the above-mentioned criteria, the parameter set was assign zero weight and HIV was not introduced for that parameter set.

**Phase 2 - HIV Prevalence Calibration**

Once all parameter sets that satisfied the behavioral data restrictions (Phase 1) were identified and HIV was introduced to each individual run, it was important to determine how well the epidemic curve produced by the HIV-CDM for each parameter set fit the UNAIDS data (Phase 2). After we produced a HIV prevalence curve for a parameter set, the model identified the section of the HIV prevalence curve that best fit the UNAIDS HIV prevalence data from 1990-2002 , by “sliding” the HIV output prevalence along the UNAIDS data (on the x-axis) until the difference between the 13 time points was as small as possible. This is equivalent to identifying which 13-year period of the model output corresponded to the best fit to the UNAIDS data from 1990-2002.

To do the fit, we used the Levenberg-Marquardt algorithm (LMA), which is a standard iterative fitting procedure that seeks to minimize the value of the sum of squares, i.e. , where $y_{i}$ was the UNAIDS HIV prevalence for year *i, M* was the HIV prevalence from the model run, *t* was the number of months the model had run since the end of model initialization and *a* was the fit parameter representing the number of months we shifted the model run to the UNAIDS HIV prevalence data points. From the fitting procedure we extracted two pieces of information: a list of the rankings of the best fitting runs based on the LMA weights and for each run, the model time points corresponding the period that provided the best fit to the 1990-2002 UNAIDS data.

**Phase 3- Reality Checks and Likelihood Weighting**

Once the prevalence output for each parameter set had been calibrated to UNAIDS data, further filtering was performed to assess whether the behavioral patterns at the end of the calibration period (2002) were as expected based on the literature. We analyzed the parameter set runs that had passed Phase 1 and Phase 2 calibration and determined if the outputs for each run fell within the ranges gathered from the literature. Any parameter set producing a value for any quantity that fell outside these ranges had its weight set to zero and was not considered further.

For the parameter sets that passed the preceding steps (thus had not had zero weights assigned), pseudo-likelihood weights were produced for each to indicate how well it fit the UNAIDS HIV prevalence data being used for calibration ([44](#_ENREF_44)). Since we ran the model for 50 years with HIV and needed at least 12 years for the curve to fit the years from 1990 to 2002, runs that took longer than 38 years to produce an epidemic were assigned zero weight; although their fit would have led to very low weights in any case, we chose to set them to zero to reduce computational time in future work.

The likelihood for run *j* that passed Phases 1, 2 and 3 of calibration was given by:

where *X_ij_* is the HIV prevalence of year *i* from run *j*, is the high estimate for the UNAIDS HIV prevalence for year i, is the low end estimate for the UNAIDS HIV prevalence for year i, Y_i_ is the UNAIDS HIV prevalence of year i, and σ is an adjustment factor that accounts for the uncertainty in the UNAIDS high and low estimates. We initially assumed σ = 1 but were able to adjust this according to observed output, as explained below.

We normalized the weights such that for run j:

We compared the calculated likelihood weights for each run against the run’s fit to evaluate whether factor adjustments were necessary. As expected, when the adjustment factor was increased, we observed a wider spread in the likelihood weights, allowing more runs to contribute to the fit of the HIV prevalence curve. After examining the effects of varying the adjustment factor, σ, across a wide range of values (0-2.5), we decided that σ=1 was the best choice since with this value the runs that contributed 90% of the weight approximated the UNAIDS curve fell almost entirely within the upper and lower UNAIDS estimates. Of the 3,750 runs that passed the calibration procedure with nonzero weights, the best-fitting 564 runs contributed 90% of the weight.

**References**

1. U.S Census Bureau. International Data Base Demographic Overview for South Africa 2012 [Available from: <http://www.census.gov/population/international/data/idb/informationGateway.php>.

2. Pettifor AE, Rees HV, Kleinschmidt I, Steffenson AE, MacPhail C, Hlongwa-Madikizela L, et al. Young people's sexual health in South Africa: HIV prevalence and sexual behaviors from a nationally representative household survey. AIDS. 2005;19(14):1525-34.

3. Shisana O, Simbayi LC. Nelson Mandela/HSRC Study of HIV/AIDS: South African National HIV Prevalence, Behavioural Risks and Mass Media: Household Survey 2002. Cape Town: Human Sciences Research Council Publishers; 2002.

4. Department of Health. South Africa Demographic and Health Survey 1998 - Full Report. 2002.

5. Connolly C, Simbayi LC, Shanmugam R, Nqeketo A. Male circumcision and its relationship to HIV infection in South Africa: results of a national survey in 2002. S Afr Med J. 2008;98(10):789-94.

6. Rain-Taljaard RC, Lagarde E, Taljaard DJ, Campbell C, MacPhail C, Williams B, et al. Potential for an intervention based on male circumcision in a South African town with high levels of HIV infection. AIDS Care. 2003;15(3):315-27.

7. Lurie MN, Williams BG, Zuma K, Mkaya-Mwamburi D, Garnett GP, Sweat MD, et al. Who infects whom? HIV-1 concordance and discordance among migrant and non-migrant couples in South Africa. AIDS. 2003;17(15):2245-52.

8. Ndase P, Celum C, Thomas K, Donnell D, Fife KH, Bukusi E, et al. Outside sexual partnerships and risk of HIV acquisition for HIV uninfected partners in African HIV serodiscordant partnerships. J Acquir Immune Defic Syndr. 2012;59(1):65-71.

9. Statistics South Africa. Marriages and divorces 2007. Statistical Release P0307. 2008.

10. Skoler-Karpoff S, Ramjee G, Ahmed K, Altini L, Plagianos MG, Friedland B, et al. Efficacy of Carraguard for prevention of HIV infection in women in South Africa: a randomised, double-blind, placebo-controlled trial. Lancet. 2008;372(9654):1977-87.

11. Gray RH, Wawer MJ, Brookmeyer R, Sewankambo NK, Serwadda D, Wabwire-Mangen F, et al. Probability of HIV-1 transmission per coital act in monogamous, heterosexual, HIV-1-discordant couples in Rakai, Uganda. Lancet. 2001;357(9263):1149-53.

12. Wawer MJ, Gray RH, Sewankambo NK, Serwadda D, Li X, Laeyendecker O, et al. Rates of HIV-1 transmission per coital act, by stage of HIV-1 infection, in Rakai, Uganda. J Infect Dis. 2005;191(9):1403-9.

13. Meekers D. Going underground and going after women: trends in sexual risk behaviour among gold miners in South Africa. Int J STD AIDS. 2000;11(1):21-6.

14. Maharaj P, Cleland J. Risk perception and condom use among married or cohabiting couples in KwaZulu-Natal, South Africa. Fam Plann Perspect. 2005;31(1):24-9.

15. Jewkes R, Vundule C, Maforah F, Jordaan E. Relationship dynamics and teenage pregnancy in South Africa. Soc Sci Med. 2001;52(5):733-44.

16. Hargreaves JR, Bonell CP, Morison LA, Kim JC, Phetla G, Porter JD, et al. Explaining continued high HIV prevalence in South Africa: socioeconomic factors, HIV incidence and sexual behaviour change among a rural cohort, 2001-2004. AIDS. 2007;21(7 Suppl):S39-48.

17. Magnani R, Macintyre K, Karim AM, Brown L, Hutchinson P, Kaufman C, et al. The impact of life skills education on adolescent sexual risk behaviors in KwaZulu-Natal, South Africa. J Adolesc Health. 2005;36(4):289-304.

18. Karim QA, Karim SS, Soldan K, Zondi M. Reducing the risk of HIV infection among South African sex workers: socioeconomic and gender barriers. Am J Public Health. 1995;85(11):1521-5.

19. CASCADE Collaboration. Differences in CD4 cell counts at seroconversion and decline among 5739 HIV-1-infected individuals with well-estimated dates of seroconversion. J Acquir Immune Defic Syndr. 2003;34(1):76-83.

20. Mills E, Cooper C, Anema A, Guyatt G. Male circumcision for the prevention of heterosexually acquired HIV infection: a meta-analysis of randomized trials involving 11,050 men. HIV Med. 2008;9(6):332-5.

21. Auvert B, Taljaard D, Lagarde E, Sobngwi-Tambekou J, Sitta R, Puren A. Randomized, controlled intervention trial of male circumcision for reduction of HIV infection risk: the ANRS 1265 Trial. PLoS Med. 2005;2(11):e298.

22. Gray RH, Kigozi G, Serwadda D, Makumbi F, Watya S, Nalugoda F, et al. Male circumcision for HIV prevention in men in Rakai, Uganda: a randomised trial. Lancet. 2007;369(9562):657-66.

23. Bailey RC, Moses S, Parker CB, Agot K, Maclean I, Krieger JN, et al. Male circumcision for HIV prevention in young men in Kisumu, Kenya: a randomised controlled trial. Lancet. 2007;369(9562):643-56.

24. Weller S, Davis K. Condom effectiveness in reducing heterosexual HIV transmission. Cochrane Database Syst Rev. 2002(1):CD003255.

25. Shisana OR, T. Simbayi, LC. Zuma, K. Jooste, S. Zungu, N. Labadarios, D. Onoya D. et al. South African National HIV Prevalence, Incidence, and Behavioral Survey, 2012. Cape Town; 2014.

26. April MD, Walensky RP, Chang Y, Pitt J, Freedberg KA, Losina E, et al. HIV testing rates and outcomes in a South African community, 2001–2006: implications for expanded screening policies. J Acquir Immune Defic Syndr. 2009;51(3):310.

27. Williams BG, Korenromp EL, Gouws E, Schmid GP, Auvert B, Dye C. HIV infection, antiretroviral therapy, and CD4+ cell count distributions in African populations. J Infect Dis. 2006;194(10):1450-8.

28. National Department of Health. The South African National Antiretroviral Treatment Guidelines 2013.

29. Barth R, van der Loeff M, Schuurman R, Hoepelman A, Wensing A. Virological follow-up of adult patients in antiretroviral treatment programmes in sub-Saharan Africa: a systematic review. Lancet Infect Dis. 2010;10(3):155-66.

30. Rosen S, Fox MP, Gill CJ. Patient retention in antiretroviral therapy programs in sub-Saharan Africa: a systematic review. PLoS Med. 2007;4(10):e298.

31. Fox MP, Rosen S. Patient retention in antiretroviral therapy programs up to three years on treatment in sub‐Saharan Africa, 2007–2009: systematic review. Trop Med Int Health. 2010;15(s1):1-15.

32. Mellors JW, Muñoz A, Giorgi JV, Margolick JB, Tassoni CJ, Gupta P, et al. Plasma viral load and CD4+ lymphocytes as prognostic markers of HIV-1 infection. Ann Int Med. 1997;126(12):946-54.

33. National Technical Information Service. Multicenter AIDS cohort study (MACS) public dataset: release P12. Springfield, VA: National Technical Information Service; 2004.

34. Boily MC, Baggaley RF, Wang L, Masse B, White RG, Hayes RJ, et al. Heterosexual risk of HIV-1 infection per sexual act: systematic review and meta-analysis of observational studies. Lancet Infect Dis. 2009;9(2):118-29.

35. Powers KA, Poole C, Pettifor AE, Cohen MS. Rethinking the heterosexual infectivity of HIV-1: a systematic review and meta-analysis. Lancet Infect Dis. 2008;8(9):553-63.

36. South Africa S. Mid-Year Population Estimates 2013, 2014, 2015. Accessed: 20 April 2016. Available from: <http://beta2.statssa.gov.za/>.

37. UNAIDS. AIDSinfo Online Database - South Africa 2012 [Available from: <http://www.unaids.org/en/dataanalysis/datatools/aidsinfo/>.

38. Adam MA, Johnson LF. Estimation of adult antiretroviral treatment coverage in South Africa. S Afr Med J. 2009;99(9):661-7.

39. Levi J, Raymond A, Pozniak A, Vernazza P, Kohler P, Hill A. Can the UNAIDS 90-90-90 target be achieved? A systematic analysis of national HIV treatment cascades. BMJ Glob Health. 2016;1(2):e000010.

40. Takuva S, Brown AE, Pillay Y, Delpech V, Puren AJ. The continuum of HIV care in South Africa: implications for achieving the second and third UNAIDS 90-90-90 targets. AIDS. 2017;31(4):545-52.

41. Shelton JD, Halperin DT, Nantulya V, Potts M, Gayle HD, Holmes KK. Partner reduction is crucial for balanced "ABC" approach to HIV prevention. BMJ. 2004;328(7444):891-3.

42. Saphonn V, Parekh BS, Dobbs T, Mean C, Bun LH, Ly SP, et al. Trends of HIV-1 seroincidence among HIV-1 sentinel surveillance groups in Cambodia, 1999-2002. J Acquir Immune Defic Syndr. 2005;39(5):587-92.

43. Eyawo O, de Walque D, Ford N, Gakii G, Lester RT, Mills EJ. HIV status in discordant couples in sub-Saharan Africa: a systematic review and meta-analysis. Lancet Infect Dis. 2010;10(11):770-7.

44. UNAIDS. AIDSinfo Online Database - South Africa 2012 [Available from: <http://www.unaids.org/en/dataanalysis/datatools/aidsinfo/>.
